# Supplementary material for: Uptake of Human Papilloma Virus vaccine among young women living in fishing communities in Wakiso and Mukono districts, Uganda
Source: PLOS Glob Public Health. 2024 Apr 18;4(4):e0003106. doi: 10.1371/journal.pgph.0003106 (PMC11025760; doi:10.1371/journal.pgph.0003106)
Supplement: Attachment — Submitted filename: Response to reviewers.pdf [file pgph.0003106.s001.pdf]

MUTEEBWA LABAN

P. O. BOX 500269, Kampala Uganda

Email: mutlabans@gmail.com

12th January 2024.

To: The Editor-In-Chief

PLOSE Global Public Health

Dear Sir/ Madam

RE: Response to Comments in regard to the Manuscript titled "Uptake of Human Papilloma Virus vaccine among young women living in fishing communities in Wakiso and Mukono districts, Uganda".

Following the review of the above mentioned manuscript, the reviewers raised some comments to address.

We have addressed these comments point by point as described below. The cited line numbers correspond to the Revised Manuscript with tracked changes

Reviewer #1: This study investigated HPV vaccination uptake in fishing villages of Uganda in order to understand if uptake was as high as nationally reported; and factors associated with uptake. This data is important in understanding how vaccination uptake may vary across a country. There are some significant concerns with the analyses conducted and interpretations provided. I have detailed these and other comments below:

Introduction:

Please check the sentence on line 59-60. High mobility in young women limits access to health services? Is this correct?

In fishing communities, the main economic activity is fishing. Given the seasonal nature of this activity, community members often have to shift to other places which are deemed to have fish in a season. Adding to the fact that health care services are limited in these communities, shifting to further remote areas makes it more difficult to access health care like vaccination, screenings for diseases and treatment. This has been demonstrated severally in other HIV related studies we have conducted in these communities, for which one of the references are cited in-text.

Line 70: MoH recommends 2 doses of which HPV vaccine? This an important detail.

Sentence improved to add detail

Methods:
The currently stated analyses is fine but I am wondering why the authors did not conduct a logistic regression to determine which factors were associated with uptake (did vs. did not get the vaccine). It is my understanding that they know who did and did not take the vaccine (via self-report), as state in the Line 92: inclusion of those "who had a response". Would that not mean they also had those that reported no?

It is indeed true that the independent variable (Uptake of HPV) was recorded as a binary outcome (those who self-reported to have received the vaccine versus those who did not receive the vaccine). Given the binary nature of the outcome in a cross sectional study, the logistic regression would be only appropriate if the outcome is rare (<10%) where in such cases the odds ratios (measure of association estimated in the logistic regression) approximate the prevalence ratios. The alternative approaches to model binary outcomes to give the prevalence ratios as the measure of association include a log-binomial and modified Poisson regression models. Both give close estimates of the prevalence ratios. Modified Poisson regression was chosen because some log-binomial models failed to converge. Furthermore, the choice of using the survey methods to fit modified Poisson regression model was to adjust for clustering that might be introduced by the multistage sampling design. The linearized standard errors adjust for this clustering using the survey methods.

Results:
It would be beneficial to not the number of women who reported taking the vaccine, not just the percentage. This could be added to Table 2 or mentioned with the percentage on line 153.
The number of women who reported receiving HPV vaccine was added (see line 155)

The interpretation you provided starting at Line 157 appears to be an interpretation of logistic regression of the whole study population. Per Table 2, it appears you only modeled those that answered "yes" to vaccination—meaning your interpretation cannot indicate factors associated with uptake without a "control" or "negative" group. If I'm misunderstanding then you need to add the same data under "HPV uptake" for that that answered "no" either in Table 2 or Table 1.
With reference to the operational definition of HPV uptake in line 105-107, it is clear that the HPV uptake was a binary outcome where those that reported to have received the

vaccine have the outcome of interest while those without the outcome didn't receive HPV vaccine. In Table 2, we presented those who self-reported to have received the HPV vaccine per each category of the independent variables to guide the readers on how crude prevalence ratios were computed (prevalence in one group divided by prevalence in the referent group). The proportion of those without the outcome would be the inverse of the proportion of those with the outcome.

Based on the reported N for "other businesses" in occupation I suggest creating one more category breaking this up.
The same comment for Religion, especially because in the Discussion you indicate Muslim religion tends to differ in uptake.

The decision to merge the levels of these variables together was because of the limited sample size (other levels would have small frequencies) that would complicate the analysis

Table 2: The percentages in the "HPV uptake" column do not make sense. For example (age), 5 is not 20.8% of 10 (the total at the top).
The percentage reported corresponds to the uptake of HPV vaccine in each category of independent variables. The operational definition of HPV uptake is on line 105-107. For example in age group 9-19 the frequency reported in Table 1 s 24 of which only 5 had been vaccinated. Therefore uptake of HPV vaccine is 5/24 = 20.8%.

For age group of 20-25 years frequency is 70 and only 5 had received HPV vaccine. Thus 5/70 = 7.1%

With the 9-25 age group being the reference the prevalence ratio is 7.1/20.8 = 0.34 (crude prevalence ratio). Therefore, this kind of computation was done to aid readers to visualize the HPV uptake in the different levels of independent variables and easily see how prevalence ratios were computed.

Discussion:
Line 172-175: I suggest removing or softening this statement. This is a strong claim considering your data is self-reported, from only 2 communities in Uganda, and was conducted at one time point. The WHO's target is a global target and many other areas have much higher vaccination uptake.

The statement was deleted

Line 185-187: Could the difference simply represent a difference of uptake across regions in Uganda? There does not have to be a methdological reason...it could simply be fact that practices differ across Uganda.

Much as there might be regional differences in Uptake of HPV vaccines, it is also true that the adolescent population at adolescent clinics (where some studies got the study population) may be more likely to be vaccinated and have better health seeking behaviors compared to the community-wide population of young people especially from remote areas like fishing communities. Also important to note that similar studies that enrolled a community-wide population in Eastern and Northern Uganda reported similar low uptake of HPV vaccines (see line 177-178)

Line 195-197: You mention age of vaccination in Uganda for the women you targeted. I wonder, Is the vaccine strategy still the same in Uganda now as when these women would've been targeted for vaccination? Perhaps your data suggest expansion of the ages targeted.
The vaccine strategy in Uganda is still the same age 9-13 years (with an assumption that they have not had their sexual debut and thus without HPV infection). The age range in this study considered the period since HPV was introduced in Uganda in 2008. The girls who had a maximum eligible age of 13 years in 2008 would be 25 years old in 2020- the year when the study was conducted.

Limitations: Self-report is a large issue here and should be mentioned specifically.
Another is the fact that this data comes from only 2 communities in Uganda and is not representative of the whole country or even other communities.

Thank you for raising this. We had missed it and we acknowledge that it is a significant limitation. We have included it in the limitation section

Line 217-221. This is not clear—potential confounders were not measured and so controlled for? Is this a typo? Also how do you know the biases did not influence study findings substantially--there was no bias analyses and many factors were not able to be accounted for.

The typo in the sentence has been corrected (unmeasured confounders were not controlled for)

Much as quantitative bias analysis was not done, some important strategies were put in place to reduce the influence of bias. For example, we report on the sensitivity analysis of the participants with missing data on outcome status that were excluded. The sensitivity analysis showed that those that were excluded were not significantly different from those that were included in the analysis. Secondly the confidence intervals of the estimates don’t appear too wide which reflects on the less impact of random error (imposed by a small sample size).

Reviewer #2: Comments to the Author:
Thanks for giving the opportunity to review the manuscript. We suggest that the paper has great potential to be published in this journal.

The article addresses an important public health issue – the low uptake of the Human Papillomavirus (HPV) vaccine among young women in fishing communities in Uganda. The study provides relevant background information, **methods, results, and discussions. Below are some specific points for consideration:**

Thank you

1. Title: Consider making the title more specific by including information about the fishing communities or emphasizing the focus on socio-demographic factors. For example, " Unraveling HPV Vaccine Uptake Challenges: Insights from Fishing Communities in Uganda" or "Low Uptake of HPV Vaccine in Fishing Communities: A Socio-demographic Analysis in Wakiso and Mukono Districts, Uganda."
Much as we present the assessment of socio-demographic factors that predict HPV Vaccination uptake, the overarching goal was just to assess the uptake of HPV vaccines among young women (who are also at an elevated risk of getting HIV). Therefore the study tittle was formulated in such a way that reflects on the overarching goal.

2. Abstract: Clarify the term "primary implementation study" in the abstract. Provide a brief explanation or definition for readers who may not be familiar with the term.
The "primary implementation study" has not special meaning apart from the fact that this study was a secondary data analysis of the "primary study" data for which the overall objective is stated in the manuscript. I hope this clarifies that the primary/parent study is what is being referred to.

3. Introduction: Provide a broader context of the HPV vaccination landscape in Uganda. Discuss any existing challenges, successes, or trends in HPV vaccine uptake nationally or in similar communities.

The Ugandan context is discussed in the introduction line 67-77

4. Methods: Consider breaking down the methods section into subsections (e.g., Study Design, Participants, Data Collection, Data Analysis) to enhance clarity and organization.

The information on study design, participants, data collection and data analysis are all covered in the subsections presented in the methods section.

For example Study design is presented under section “study design and setting” line 81-91. The participants are covered under the sub-section “study population, inclusion and exclusion criteria" line 92-98. Data collection is covered under sub-section “sampling and data collection” line 99-103. Data analysis covered under sub-section “Data analysis” line 112-129.

5. Results: Include tables or graphs to visually present key results, such as the socio-demographic characteristics of study participants, age distribution, and HPV vaccine uptake rates.
All study results are presented in tables except for the sensitivity analysis that is not presented in this paper but was done to assess the impact of missing data

6. Discussion: Comparison with Other Studies: Compare the findings of this study with those of similar studies in Uganda or other regions to provide a broader perspective on HPV vaccine uptake.

Study findings were compared to other Ugandan studies describing uptake of HPV vaccines in the other regions (see line 177-179 and line 185-186) .

7. Discuss potential reasons for the low HPV vaccine uptake observed in fishing communities, such as access barriers, awareness issues, or community-specific challenges.

Potential reasons for low uptake of HPV vaccines in fishing communities discussed. Update made from line 189-193

8. Propose more detailed strategies for improving HPV vaccine uptake in fishing communities based on the study findings. This could include community engagement programs, targeted awareness campaigns, or collaboration with religious leaders.

These have been included

9. Explicitly acknowledge potential biases in the limitations section and discuss how these biases may have influenced the study results.

Potential biases are discussed under limitation section. This section has been updated to acknowledge self-report nature of outcome, recall bias, missing data, and small sample size

10. Condense the recommendations section to emphasize key points. Clearly outline the proposed interventions for improving HPV vaccine uptake.

Recommendations updated to include proposed interventions for improving HPV

vaccine uptake

Overall Impression: The study addresses a significant public health concern and provides valuable insights into the factors influencing HPV vaccine uptake. Enhancements in presentation, clarity, and expansion of certain sections could further strengthen the article. Overall, the work contributes to the understanding of vaccination challenges in specific populations and highlights the need for targeted interventions.

Thank you

We thank you so much for the very insightful and valuable comments that were provided aimed to improve the quality of the paper.

We look forward to your feedback, and we are happy to respond to any other comments that come up

Thank you again for considering this manuscript under your review.

Sincerely

Muteebwa Laban
