# Supplementary material for: Uptake of Human Papilloma Virus vaccine among young women living in fishing communities in Wakiso and Mukono districts, Uganda
Source: PLOS Glob Public Health. 2024 Apr 18;4(4):e0003106. doi: 10.1371/journal.pgph.0003106 (PMC11025760; doi:10.1371/journal.pgph.0003106)
Supplement: Attachment — Submitted filename: Response to reviewers.pdf [file pgph.0003106.s002.pdf]

Response to reviewers

Please revise table 2. It is unclear and there is no need for p-values and CI. Also, the response did not sufficiently address the prior reviews concerns. Please revise.

The previous reviewer's comments:

Table 2: The percentages in the "HPV uptake" column do not make sense. For example (age), 5 is not 20.8% of 10 (the total at the top

Table 2: I suggest make the reference groups in the analyses so that all PRs are >1. This simplifies interpretation for the reader.

Response: Table 2 has been updated so that all PRs are >1. Column HPV uptake has been deleted. Much as both confidence intervals and P values draw the same conclusion on interpretation, both the confidence intervals and P values were presented. This is because the confidence intervals facilitate the examination of the interval and assessing the role of random error. Also because we used a statistical approach (P values <0.2) to select variables to include in the multivariate analysis, the P values were also presented
